# Exotic Spectra and Lattice Vibrations of Ice X Using the DFT Method

**DOI:** 10.3390/molecules23112780

**Published:** 2018-10-26

**Authors:** Lu Jiang, Shu-Kai Yao, Kai Zhang, Ze-Ren Wang, Hui-Wen Luo, Xu-Liang Zhu, Yue Gu, Peng Zhang

**Affiliations:** School of Space Science and Physics, Shandong University, Weihai 264209, China; Jiang_Lu@163.com (L.J.); yao201@purdue.edu (S.-K.Y.); 17862711589@163.com (K.Z.); wangzeren96@163.com (Z.-R.W.); luohuiwen1900@163.com (H.-W.L.); zhuxuliang@outlook.com (X.-L.Z.); du_guyue@163.com (Y.G.)

**Keywords:** ice X, vibrational spectrum, first-principles DFT, Raman scattering, IR absorption

## Abstract

A typical vibrational spectrum in the ice phase has four separate bands: Translation, libration, bending, and stretching. Ice X, the final ice phase under high pressure, shows an exotic vibrational spectrum. Based on harmonic approximation, an ideal crystal of ice X has one peak, at 998 cm^−1^, for Raman scattering and two peaks, at 450 cm^−1^ and 1507 cm^−1^, for infrared absorption in this work. These three characteristic peaks are indicators of the phase transition between ice VII and VIII and ice X. Despite many experimental and theoretical works on ice X, only this study has clearly indicated these characteristic peaks in the region of the IR band. The phonon density of states shows quite different features than ice VIII, which could be verified by inelastic neutron scattering in the future. The dynamic processes of 15 vibrational normal modes are discussed and the typical hydrogen bonds are missing.

## 1. Introduction

Water is one of the most abundant materials. It is important for both nature’s balance and human evolution. It is essential in both Earth and planetary sciences to understand the behavior of water over a broad range of thermodynamic conditions and experiments [1]. As the solid state of water, ice has been found in at least 20 formations of crystalline and amorphous phase structures under different temperature and pressure conditions [2,3,4,5].

As early as 1972, Holzapfel was the first to predict a high-pressure phase of ice that exists with hydrogen-bonded protons residing in the middle of two symmetrically neighboring oxygen atoms [6]. In 1984, after reporting the results of a Brillouin scattering experiment on solid ice up to 30 GPa, Polian et al. presented an extension of those results up to 67 GPa [7], which also predicted the new high-pressure phase (i.e., ice X). Under such high pressure, the orientations of ice X are ordered and its hydrogen atoms lie at symmetric midpoints, leading to the reasonable prediction that the structure is ionic in nature. Despite the different space groups of ice VIII and ice X, their atomic arrangements and lattice constants are very similar. As such, it has been predicted that ice VIII can become ice X under certain pressures and temperatures [8]. Using infrared (IR) measurements, Goncharov et al. provided evidence of the phase transition from ice VII and VIII to ice X, beginning at 60 GPa and reaching a stable stage of at least 210 GPa [9]. They identified one peak at approximately 1500 cm^−1^ at pressure above 175 GPa as an indicator of ice X. Their calculations indicate that there are two IR absorption peaks at 1508 cm^−1^ and 450 cm^−1^ at pressure above 120 GPa.

Two symmetric O–H bonds connect each hydrogen atom in the ice X crystal. Consequently, it is no longer in a molecular phase, but in an atomic one [6]. Considerable investigations have been conducted on the physical and chemical properties of ice X [10,11,12,13,14]. However, no inelastic neutron scattering (INS) experiment has been reported under such high pressure. Marqués et al. conducted a first-principles study of phonon frequencies [11]. Their density functional theory (DFT) calculations illustrate that ice X is not molecular in character and is linked by soft phonon transitions to ice VIII (or disordered ice VII) at low pressure and to a putative *Pn*3*m* structure at high pressure [11]. In addition, the calculated phonon frequencies in their work indicate that ice X is a stable phase, but with the pressure decreasing below 110 GPa, the dispersion curve changes significantly. Putrino and Parrinello theoretically reported a Raman peak at 840 cm^−1^ [12]. Alexander [13] and Men [14] observed this peak experimentally at 980 cm^−1^ and 785 cm^−1^. However, a unique Raman peak for ice X has not been proposed. In this study, to clarify the critical indicators of the transition from ice VIII to X, 15 normal vibration modes of ice X are individually analyzed and three vibrational spectra are illustrated for reference to the phase transition.

## 2. Computational Methodology

**Figure 1:**
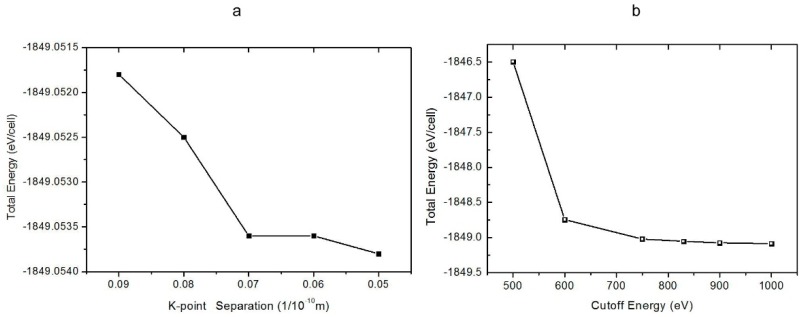
Diagram of the k-point and energy cutoff convergence tests. The total energy against k-point separation is shown in (**a**) and the cutoff convergence is shown in (**b**). The parameters used in this study were 0.07/Å for the k-point mesh and 830 eV for the energy cutoff.

Using CASTEP [15], a first-principles DFT code, we set up a route map to calculate the geometry and lattice vibrations of the ice X phase. The conventional cell of ice VIII, containing eight molecules, was built as a prototype, space group *I*4_1_/*amd*. As shown in Figure 1, we first conducted k-point and energy cutoff convergence tests. As a phase transition occurred in the process of calculation, we defined the k-point separation instead of the k-point mesh. Figure 1a shows that the separation of 0.07/Å was sufficient. We set 830 eV as the energy cutoff. Although not a big value, it was appropriate for this study, as shown in Figure 1b. A hydrostatic pressure, ranging from 70 GPa to 140 GPa, was applied with a 10 GPa interval. The hydrogen gradually shifted at the midpoint between the two oxygens as the pressure increased. The structure eventually fully transformed into space group *Pn*3*m* at 120 GPa. Then, the phonons and IR properties of ice X were calculated to simulate the phonon density of states (PDOS) and the photon scattering spectra at this pressure. The normal vibration modes were extracted from the property calculation of the polarizability, IR, and Raman spectra. The exchange–correlation function of the generalized gradient approximation RPBE [16] was selected for geometrical optimization. As the hydrogen bonding of the RPBE was slightly underestimated, the subsequent vibrational spectrum calculations presented a small blue shift. The energy and SCF tolerance were taken as 1 × 10^−9^ for the PDOS calculation with norm-conserving pseudopotentials.

## 3. Results and Discussion

**Figure 2:**
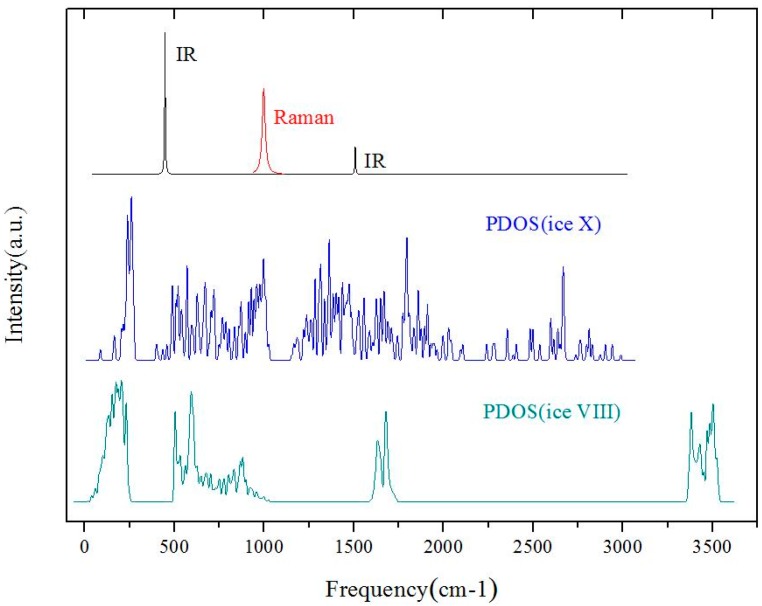
Three simulated spectra of ice X. IR, Raman, and the phonon density of states (PDOS) are colored in black, red, and blue, respectively. The PDOS curve of ice VIII is shown at the bottom for comparison.

Figure 2 illustrates the three simulated spectra (i.e., Raman, IR, and INS) of ice X. For comparison, the PDOS of ice VIII is also listed at the bottom. As for the PDOS of ice X, the spectrum could not be divided into four separate regions as with ice VIII. In the ice family, the intramolecular O–H stretching region occurred above 3000 cm^−1^. There were no frequencies at this region for ice X, meaning that strong covalent bonds were missing. The PDOS peaks of ice X below 300 cm^−1^ were from the acoustic branch and the band from 400 cm^−1^ to 1000 cm^−1^ was from the optic branch. Obviously, the intermolecular hydrogen bonds were also missing. These features at above 400 cm^−1^ were similar to the librational bands of other ice phases. However, the stubborn isolated intramolecular O–H bending peaks, at approximately 1600–1700 cm^−1^, extended to a broad band. On account of the high pressure, ice X demonstrated metallic properties to some extent. It is unsurprising that the spectrum of ice X was quite different from the spectra of traditional ice phases. The PDOS curve reflected phonons integrated by the entire reduced Brillouin zone. As the signals collected by the INS experiment were proportional to the PDOS, this simulated curve may be verified via INS in the future. In spite of its PDOS complexity, the IR and Raman spectra showed very distinct indicators of ice X identification. Our simulations showed that two peaks, at 450 cm^−1^ and 1508 cm^−1^, were IR active and that only one peak, at 998 cm^−1^, was Raman active (A_1g_ and E_g_ were Raman active, and T_1u_ was IR active). In the IR study of Goncharov et al., it was obvious to assign a peak at approximately 1500 cm^−1^ in the experimental spectrum [9]. However, the lower peak was not reported. Putrino and Parrinello predicted a Raman peak at 840 cm^−1^ [12]. However, there were still other Raman peaks in their theoretical spectrum. Goncharov [13] and Men [14] observed this Raman peak at 980 cm^−1^ and 785 cm^−1^, respectively. As vibrational frequencies are very sensitive to pressure, big differences in peak position may reflect various pressure conditions. For the first time, we theoretically confirm three indicator peaks in ice X, one for Raman scattering and the other two for IR absorption.

The periodic structure of ice X is a six-atom primitive cell, which greatly reduces the normal vibrational modes. Ice X has 3 × 6 − 3 = 15 optic modes. The 15 modes are discussed individually below.

**Figure 3:**
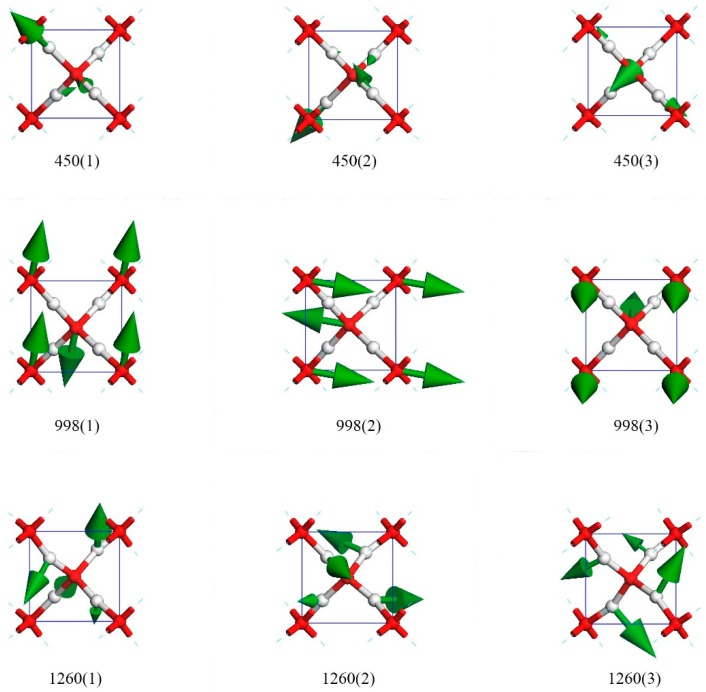
Nine normal vibrational modes at 450 cm^−1^, 998 cm^−1^, and 1260 cm^−1^. Each frequency has three degenerate states.

Figure 3 presents nine top views of vibrational modes in a primitive cell. The three modes in row one were degenerate states in which only hydrogen atoms vibrate. The green arrows indicate the oscillating directions and are proportional to the amplitude in size. To maintain a static center of mass, the collective vibrations were coupled. We used the direction of hydrogen’s biggest amplitude to depict these modes. For the vibration mode at 450(1), the oscillating direction of the biggest hydrogen was along (1,−1,−1). The other two crystal array indices of modes 450(2) and 450(3) were (1,1,1) and (1,1,−1), respectively. Supplementary Materials Video S1 illustrates the dynamic process in detail. Figure 2 shows that this peak was IR active. Through ab initio calculations of the Raman spectra at 125 GPa, Putrino and Parrinello showed a peak at 390 cm^−1^ [12]. However, they could not find this peak in their Raman scattering experiment [12]. We found that this peak was Raman inactive.

The three degenerate states at 998 cm^−1^ had all oxygen atoms vibrating, whereas the hydrogens remained static in the lattice. As their relative vibrational directions were opposite, we labeled the directions of oscillation to distinguish these three modes, which were roughly depicted as (0,1,0), (1,0,0), and (0,0,1). There was little deviation from these three directions. We confirm that they were three orthorhombic vibrational directions and that they were sensitive to different pressures. This was the only Raman active peak based on harmonic approximation. In their work on Raman spectra, Putrino and Parrinello [12] reported the peak at approximately 950 cm^−1^. Their experimental data indicated that the peak occured at 840 cm^−1^, which agrees with our result at 998 cm^−1^. The reported anharmonic motions cannot be calculated in this work. Supplementary Materials Video S2 illustrates the dynamic process in detail. Through his work on Raman spectra at 128 GPa, Alexander [13] also verified a dominant peak at approximately 980 cm^−1^.

There were three rotating modes at 1260 cm^−1^. These modes were similar to the twisting modes in the librational bands of other ice phases. As shown in Figure 3, the hydrogens oscillated perpendicularly to the O–O line, and the four hydrogens rotated around the central oxygen to form a vortex. The rotation axis could be roughly regarded as (1,−1,1), (1,1,1), and (1,1,−1). Supplementary Materials Video S3 illustrates the dynamic process in detail.

**Figure 4:**
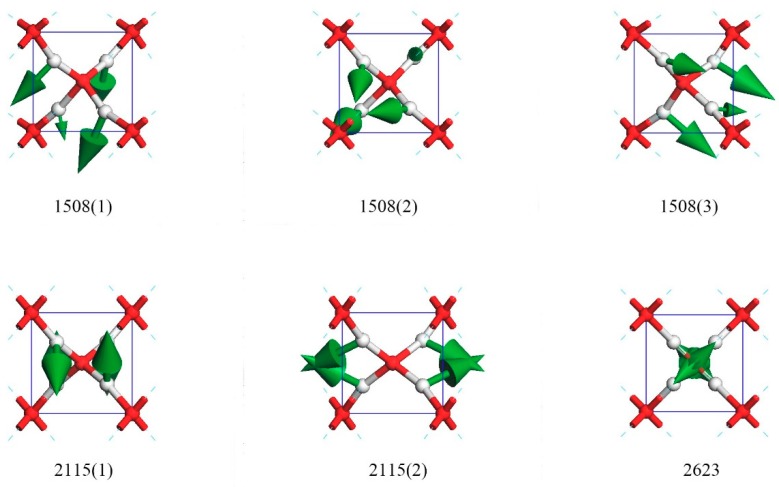
Six normal vibrational modes at 1508 cm^−1^, 2115 cm^−1^, and 2623 cm^−1^. The maximum strength at 2623 cm^−1^ was non-degenerate.

Figure 4 shows three bending modes at 1508 cm^−1^ in which the four O–H–O bonds bent in the same direction (three were strong and one was weak). These three degenerated modes composed the other peak of IR active. The three directions of degenerate modes at 1508(1), 1508(2), and 1508(3) were approximately equal to (1,1,1), (1,1,−1), and (1,−1,1), respectively. Supplementary Materials Video S4 illustrates this process.

Interestingly, there were two degenerate modes at 2115 cm^−1^ and one isolated mode at 2623 cm^−1^. In the case of 2115(1), the two hydrogen atoms oscillated in the opposite direction along (1,1,1), whereas the other two moved along (−1,1,1). For 2115(2), the mode could be regarded as one H–O–H bending in the (−1,0,0) direction, whereas the other one vibrated in the (1,0,0) direction. Vibrational frequency at 2623 cm^−1^ was a unique mode, which was the upmost frequency. In this mode, the four hydrogen atoms simultaneously vibrated toward the central oxygen. This was a symmetrical motion through the center of inversion and was, of course, IR and Raman inactive. Supplementary Materials Video S5 illustrates the dynamic process of the 2115(1), 2115(2), and 2623 modes via a movie.

## 4. Conclusions

Fifteen normal vibrational modes of ice X are discussed based on the DFT calculation. The PDOS integrated by dispersion curves showed quite complex features. However, photon scattering theoretically presented very simple features—one peak, at 998 cm^−1^, for Raman scattering and two peaks, at 450 cm^−1^ and 1508 cm^−1^, for IR absorption. These three peaks are indicators of complete ice X phase transition. Phase transition was observed above 60 GPa. We conclude that the transition region ranges from 60 GPa to 120 GPa. The complex vibrational peaks observed by experiments were from incomplete phase transition in this range. As the vibration frequencies are pressure-dependent, the pressure conditions should be examined when considering the three peaks mentioned above.

## Supplementary Materials

The following are available online, Video S1: Dynamic process of three normal modes at 450 cm^−1^; video S2: Dynamic process of three normal modes at 998 cm^−1^; video S3: Dynamic process of three normal modes at 1260 cm^−1^; video S4: Dynamic process of three normal modes at 1508 cm^−1^; and video S5: Dynamic process of two normal modes at 2115 cm^−1^ and the maximum mode at 2263 cm^−1^.
